# Critical Role for the NLRP3 Inflammasome in Mediating IL-1β Production in *Shigella sonnei*-Infected Macrophages

**DOI:** 10.3389/fimmu.2020.01115

**Published:** 2020-06-03

**Authors:** Lan-Hui Li, Tzu-Ling Chen, Hsiao-Wen Chiu, Chung-Hua Hsu, Chien-Chun Wang, Tzu-Ting Tai, Tz-Chuen Ju, Fang-Hsin Chen, Oleg V. Chernikov, Wen-Chiuan Tsai, Kuo-Feng Hua

**Affiliations:** ^1^Department of Laboratory Medicine, Linsen, Chinese Medicine and Kunming Branch, Taipei City Hospital, Taipei, Taiwan; ^2^Department of Pathology, Tri-Service General Hospital, National Defense Medical Center, Taipei, Taiwan; ^3^Department of Biotechnology and Animal Science, National Ilan University, Ilan, Taiwan; ^4^Institute of Traditional Medicine, School of Medicine, National Yang-Ming University, Taipei, Taiwan; ^5^Infectious Disease Division, Linsen, Chinese Medicine and Kunming Branch, Taipei City Hospital, Taipei, Taiwan; ^6^Department of Animal Science and Biotechnology, Tunghai University, Taichung, Taiwan; ^7^Department of Medical Imaging and Radiological Sciences, Chang Gung University, Taoyuan, Taiwan; ^8^G.B. Elyakov Pacific Institute of Bioorganic Chemistry FEB RAS, Vladivostok, Russia; ^9^Department of Medical Research, China Medical University Hospital, China Medical University, Taichung, Taiwan

**Keywords:** shigellosis, NLRP3 inflammasome, macrophages, P_2_X_7_ receptor, mitochondria

## Abstract

*Shigella* is one of the leading bacterial causes of diarrhea worldwide, affecting more than 165 million people annually. Among the serotypes of *Shigella, Shigella sonnei* is physiologically unique and endemic in human immunodeficiency virus-infected men who have sex with men. The NOD-, LRR-, and pyrin domain-containing protein 3 (NLRP3) inflammasome, a protein complex composed of NLRP3, apoptosis-associated speck-like protein, and caspase-1, recognizes, and responds to pathogen infection and diverse sterile host-derived or environmental danger signals to induce IL-1β and IL-18 production. Although the *Shigella flexneri*-mediated activation of the NLRP3 inflammasome has been reported, the effect of *S. sonnei* on NLRP3 inflammasome activation remains unclear. We found that *S. sonnei* induced IL-1β production through NLRP3-dependent pathways in lipopolysaccharide-primed macrophages. A mechanistic study revealed that *S. sonnei* induced IL-1β production through P_2_X_7_ receptor-mediated potassium efflux, reactive oxygen species generation, lysosomal acidification, and mitochondrial damage. In addition, the phagocytosis of viable *S. sonnei* was important for IL-1β production. Furthermore, we demonstrated that NLRP3 negatively regulated phagocytosis and the bactericidal activity of macrophages against *S. sonnei*. These findings provide mechanistic insight into the activation of the NLRP3 inflammasome by *S. sonnei* in macrophages.

## Introduction

Shigellosis is a bacillary dysentery caused by Gram-negative, non-motile rod-shaped *Shigella* species. *Shigella* infects the intestines of humans and higher primates, resulting in acute diarrhea that may contain blood and mucus (1). Globally, there were at least 26 million cases of shigellosis from 1990 to 2016 and 212,438 deaths in 2016 (2). Each year, ~500,000 cases of diarrhea and 40 deaths caused by *Shigella* are reported in the United States (3). In Taiwan, there were 172 notifiable cases in 2018 according to a report by the Center of Disease Control (Taiwan CDC). *Shigella* belongs to the *Enterobacteriacae* family, which comprises four *Shigella* species, namely, *Shigella dysenteriae, Shigella flexneri, Shigella boydii*, and *Shigella sonnei*; these species are distributed worldwide, and their resistance to ciprofloxacin, ceftriaxone, and azithromycin is emerging (4, 5). Particularly in men who have sex with men (MSM) and HIV-positive populations, azithromycin-resistant *Shigella* is spreading globally (6). Outbreaks of *S. flexneri* and *S. sonnei* among MSM have been reported more frequently in the US, Canada, England, and Spain in recent years (1, 5, 7). In addition, from 2015 to 2016 in Taiwan, an outbreak of shigellosis was reported in MSM living with HIV (8). Taipei City Hospital isolated *S. sonnei* from several clinical shigellosis cases in MSM with HIV. Watery or bloody diarrhea caused by *S. sonnei* is usually relatively mild illness; however, its spread between MSM by sexual transmission is a public health concern.

*Shigella* invades and destroys the lining of the colon and the rectum mucosa and then enters resident macrophages and dendritic cells (9). Once these cells are infected, *Shigella* induces vacuole lysis, intracellular replication, and inflammatory cell death. It eventually disseminates and triggers a severe inflammatory response and cause acute bloody diarrhea (10). During shigellosis, the formation of micro-ulcers and inflammatory exudates of the colonic epithelium lead to polymorphonuclear leucocytes, and blood appears in the feces. Inflammation is a protective process that restricts microbial infection. Nucleotide-binding and oligomerization domain (NOD)-like receptor (NLR) is an intracellular innate immune receptor that recognizes and triggers inflammation against bacterial infection (11). Inflammasomes are multiprotein complexes comprised of members of the NLR family and/or apoptosis-associated speck-like protein (ASC) in response to intracellular pathogen- or damage-associated molecular patterns (12). Of the discovered inflammasomes, the NLRP3 inflammasome is the most well-investigated because it is highly relevant to human diseases (13–15).

Infection with Gram-negative bacteria from the *Enterobacteriaceae* family, such as *Salmonella typhimurium, Escherichia coli*, and *Citrobacter rodentium*, activate the NLRP3 inflammasome (16–19). In 2007, Suzuki et al., and Willingham et al., demonstrated that *S. flexneri* infection induces interleukin (IL)-1β production through Ipaf/ASC- and NLRP3/ASC-dependent pathways in macrophages (20, 21). In 2014, Suzuki et al. further determine that the *S. flexneri* type III secreted protein invasion plasmid antigen H7.8 enzyme 3 ubiquitin ligase plays pivotal role in NLRP3 inflammasome activation in macrophages (22). Although the effect of *S. flexneri* on the NLRP3 inflammasome has been well-studied, the effect of *S. sonnei*, a physiologically unique serotype of *Shigella*, on the NLRP3 inflammasome has not yet been addressed. In this study, we demonstrated the importance of the NLRP3 inflammasome in *S. sonnei*-mediated IL-1β and IL-18 production in macrophages. The roles of phagocytosis, P_2_X_7_ receptor-mediated potassium efflux, reactive oxygen species generation, lysosomal acidification, and mitochondrial damage in *S. sonnei*-mediated NLRP3 inflammasome activation were further investigated. Furthermore, the effect of NLRP3 knockout on the bactericidal activity of macrophages against *S. sonnei* was studied. This study provides evidence for the NLRP3 inflammasome as a promising drug target for *S. sonnei* infection.

## Materials and Methods

### Reagents and Chemicals

YVAD-CHO, ammonium chloride (NH_4_Cl), chloroquine diphosphate (CQ), N-acetyl cysteine (NAC), potassium chloride (KCl), glibenclamide, probenecid, carbenoxolone, LPS (*Escherichia coli* O111:B4), cyclosporine A, CA-074-Me, nordihydroguaiaretic acid (NDGA) were purchased from Sigma-Aldrich (St. Louis, MO). TLR2 shRNA lentiviral particles (sc-40257-V), control shRNA lentiviral particles (sc-108080), P_2_X_7_ shRNA plasmids (sc-42576-SH), control shRNA plasmids (sc-108060), Cryopyrin CRISPR/Cas9 KO plasmids (sc-432122), Manganese (III) tetrakis (4-benzoic acid) porphyrin chloride (MnTBAP), and antibodies against ASC (SC-22514-R, polyclonal antibody), IL-18 (SC-6177, polyclonal antibody), P_2_X_7_ (SC-514962, monoclonal antibody), and actin (SC-47778, monoclonal antibody) were purchased from Santa Cruz Biotechnology (Santa Cruz, CA). Antibodies against NLRP3 (AG-20B-0014, monoclonal antibody) and mouse caspase-1 (AG-20B-0044, monoclonal antibody) were purchased from Adipogen International (San Diego, CA). Antibodies against IL-1β (AB-401-NA, polyclonal antibody) were purchased from R&D Systems (Minneapolis, MN). MCC950 was purchased from TargetMol (Wellesley Hills, MA). DiOC_2_(3) and ELISA kits for IL-1β and tumor necrosis factor-α (TNF-α) were purchased from Thermo Fisher Scientific (Waltham, MA). Phorbol 12-myristate 13-acetate was purchased from Merck Millipore (Bedford, MA). Macrophage Colony Stimulating Factor (M-CSF) was purchased from Peprotech (London, UK).

### Cell Lines and Culture

Mouse J774A.1 macrophages and human THP-1 monocytes were purchased from the American Type Culture Collection (Rockville, MD). THP-1 macrophages were differentiated from THP-1 monocytes by treatment with 50 nM PMA for 48 h. Human peripheral blood mononuclear cells (PBMCs) were separated from whole blood from healthy volunteers by density gradient centrifugation using Histopaque-1077 (23), and all experimental protocols were performed in accordance with the guidelines and regulations provided and accepted by the Institutional Review Board of the Tri-Service General Hospital, National Defense Medical Center and the volunteers' informed consent (TSGH-IRB-2-106-05-190 and TSGH-IRB-2-106-05-009). Mouse primary bone marrow derived macrophages (BMDM) were prepared from bone marrow collected from C57BL/6 mouse femur and tibia by differentiating in the M-CSF containing medium for 7 days. Animal experiments were performed with the approval of the Institutional Animal Care and Use Committee of the National Ilan University (approval number: No. 106-13). TLR2-knockdown and scramble control J774A.1 macrophages were generated by stably infection of TLR2 shRNA lentiviral particles and control shRNA lentiviral particles, respectively. P_2_X_7_-knockdown and scramble control J774A.1 macrophages were generated by stably transfection of P_2_X_7_ shRNA plasmids and control shRNA plasmids, respectively. NLRP3-knockout J774A.1 macrophages were generated by transfection of Cryopyrin CRISPR/Cas9 KO plasmids, and the clone with significantly reduced NLRP3 protein expression was selected for further studies. All cells were cultured in RPMI-1640 medium containing 10% fetal bovine serum in a 37°C CO_2_ incubator.

### Activation of NLRP3 Inflammasome by *S. sonnei* Infection

*S. sonnei* (strain 25,931) was purchased from American Type Culture Collection. Generally, the bacteria were grown and subcultured twice a week on chocolate agar purchased from Creative Lifesciences (Taipei, Taiwan) at 37°C in 5% CO_2_. *S. sonnei* was subcultured again 1 day before infection. J774A.1 macrophages, THP-1 macrophages, or BMDM were primed for 4 h with 1 μg/ml LPS and then infected with *S. sonnei* at different multiplicities of infections (MOIs) for 1 h at 37°C. The extracellular bacteria were washed with sterile PBS and cultured in fresh medium for an additional 20 h with 3 μg/ml gentamicin (to kill the residual extracellular bacteria and avoid excessive bacterial growth in the medium). The intracellular penetration of gentamicin is low and did not have significant effect on intracellular bacteria (24). If the inhibitor (YVAD-CHO, MCC950, NH4Cl, CQ, NAC, KCl, glibenclamide, probenecid, carbenoxolone, cyclosporine A, MnTBAP, CA-074-Me, and NDGA) was used, it was added to the medium after LPS priming and 30 min before *S. sonnei* infection. The expression levels of IL-1β and TNF-α in the culture medium were measured by ELISA as described previously (25). In addition, to detect the expression levels of proIL-1β/IL-1β, proIL-18/IL-18, p45/p10, NLRP3, and ASC in the culture medium, the medium was concentrated with methanol/chloroform as described previously (26) and then analyzed by Western blotting. To detect the expression levels of proIL-1β, NLRP3, and actin in the cells, the cell lysates were analyzed by Western blotting. Heat-killed *S. sonnei* were prepared by incubating the bacteria in 80°C hot-plate for 30 min. Freeze/thaw-killed *S. sonnei* were prepared by repeatedly freezing the bacteria at −80°C for 1 h and thawing at room temperature five times. The loss of bacterial viability was confirmed by plating on a chocolate agar plate.

### Detection of Intracellular ROS

Intracellular ROS levels were measured by staining the cells with the general oxidative stress indicator CM-H_2_DCFDA (Thermo Fisher Scientific). J774A.1 macrophages were primed with 1 μg/ml LPS for 4 h and then infected with 50 MOI *S. sonnei* for an additional 20 h. Then, the cells were stained with 2 μM CM-H_2_DCFDA for 15 min, and the intracellular fluorescence intensity was detected by flow cytometry (Cytomics FC500 Flow Cytometry CXP, Beckman Coulter Life Sciences).

### Detection of Mitochondrial ROS and Membrane Potential

To detect mitochondrial ROS production, J774A.1 macrophages were primed with 1 μg/ml LPS for 4 h and then incubated with or without 20 μM MnTBAP for 30 min. The cells were infected with *S. sonnei* at 50 MOI for an additional 20 h. Then, the cells were stained with 5 nM MitoSOX for 15 min, and the fluorescence signal was acquired by flow cytometry. To detect the mitochondrial membrane potential, J774A.1 macrophages were primed with 1 μg/ml LPS for 4 h and then infected with 50 MOI *S. sonnei* for an additional 20 h. Then, the cells were stained with 50 nM DiOC_2_(3) for 15 min. The fluorescence signals were detected by flow cytometry.

### Phagocytosis and Bactericidal Activity Assay

For short-term treatment, wild-type and NLRP3 knockout J774A.1 macrophages were infected with *S. sonnei* at 50 MOI for 15 min at 37°C in a CO_2_ incubator. The extracellular bacteria were washed out with sterile PBS and incubated in PBS containing 300 μg/ml gentamicin for an additional 1 h at 37°C in a CO_2_ incubator to completely kill the extracellular bacteria. The cells were washed with PBS and lysed with 300 μl distilled water for 40 min at 37°C in a CO_2_ incubator. The lysate was diluted 300 times with PBS, and 200 μl diluted lysate was then inoculated on chocolate agar plates and incubated at 37°C in a CO_2_ incubator overnight. The number of colony-forming units (CFUs) was counted and calculated. For long-term treatment, wild-type and NLRP3 knockout J774A.1 macrophages were infected with *S. sonnei* at 50 MOI for 15 min at 37°C in a CO_2_ incubator. The extracellular bacteria were washed out with sterile PBS and cultured in medium containing 100 μg/ml gentamicin for an additional 20 h at 37°C in a CO_2_ incubator. The cells were washed with PBS and lysed in 300 μl distilled water for 40 min at 37°C in a CO_2_ incubator. The lysate was diluted 50 times with PBS, and then 200 μl diluted lysate was inoculated on chocolate agar plates and incubated at 37°C in a CO_2_ incubator overnight. The number of CFUs was counted and calculated. Bactericidal activity was presented as the number of killed bacteria, which was calculated by subtracting the long-term CFU assay result from the short-term CFU assay result.

### Statistical Analysis

Statistical significance was determined by GraphPad Prism 7.0 software. Two-tailed *t*-tests were used for two groups, and ANOVA with Dunnett's multiple comparisons test was used for three or more groups. ^*^, ^**^, and ^***^ indicate a significant difference at the levels of *p* < 0.05, *p* < 0.01 and *p* < 0.001, respectively.

## Results

### *S. sonnei* Induces the Secretion of IL-1β, IL-18, NLRP3, ASC, and Active Caspase-1 in Macrophages

**Figure 1:**
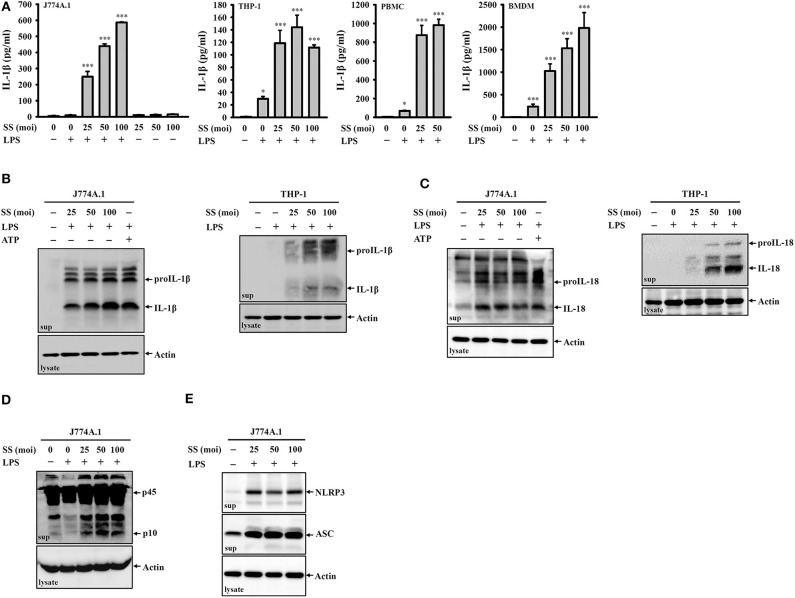
*S. sonnei* induces the secretion of IL-1β, IL-18, NLRP3, ASC, and active caspase-1 in macrophages. **(A)** J774A.1 macrophages, THP-1 macrophages, PBMCs or BMDM were primed with 1 μg/ml LPS for 4 h and then infected with *S. sonnei* for an additional 20 h. The levels of IL-1β in the supernatants were measured by ELISA. **(B–E)** J774A.1 macrophages or THP-1 macrophages were primed with 1 μg/ml LPS for 4 h followed and then infected with *S. sonnei* for an additional 20 h or stimulated with 5 mM ATP for an additional 0.5 h. The levels of IL-1β **(B)**, IL-18 **(C)**, caspase-1 **(D)**, NLRP3, and ASC **(E)** in the supernatants were measured by Western blotting. The ELISA data are expressed as the mean ± SD of four separate experiments. The Western blotting results are representative of three different experiments. * and *** indicate significant differences at the levels of *p* < 0.05 and *p* < 0.001, respectively, compared to untreated control cells (one-way ANOVA with Dunnett's multiple comparisons test).

To investigate whether *S. sonnei* infection induces IL-1β secretion, untreated or LPS-primed mouse J774A.1 macrophages were infected with *S. sonnei* at 25, 50, or 100 MOI for 24 h. We found that *S. sonnei* induced IL-1β secretion in LPS-primed J774A.1 macrophages but not in untreated J774A.1 macrophages (Figure 1A). These results indicated that *S. sonnei* provided the activation signal but not the priming signal of inflammasome in macrophages. *S. sonnei* infection also induced IL-1β secretion in LPS-primed human THP-1 macrophages, human PBMCs, and mouse BMDM (Figure 1A). The *S. sonnei*-mediated induction of IL-1β was confirmed by detecting IL-1β expression in the culture medium of J774A.1 and THP-1 macrophages by Western blotting (Figure 1B). In addition, *S. sonnei* infection increased the expression of another inflammasome product, IL-18, in the culture medium of J774A.1 and THP-1 macrophages, as analyzed by Western blotting (Figure 1C). LPS- and ATP-activated J774A.1 macrophages were a positive control for IL-1β and IL-18 secretion, because ATP strongly activates the NLRP3 inflammasome in mouse macrophages, but only slightly activates the NLRP3 inflammasome in THP-1 macrophages. As the maturation and secretion of IL-1β and IL-18 are regulated by caspase-1, we asked whether caspase-1 is activated by *S. sonnei* infection. We found that the level of active caspase-1 (p10) in the culture medium of J774A.1 macrophages was increased by *S. sonnei* infection, as analyzed by Western blotting (Figure 1D). It has been demonstrated that upon the activation of inflammasome, inflammasome components are released from macrophages, act as extracellular danger signals and amplify the inflammatory response (27). We found that *S. sonnei* infection induced NLRP3 and ASC release into the culture medium of J774A.1 macrophages (Figure 1E).

### *S. sonnei* Induces IL-1β Secretion Through the NLRP3 Inflammasome

**Figure 2:**
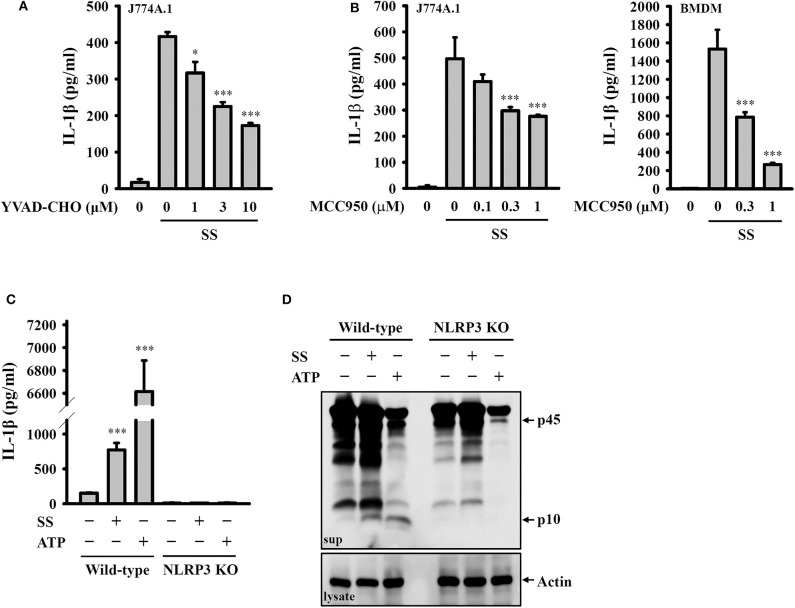
*S. sonnei* induces IL-1β secretion through the NLRP3 inflammasome. **(A,B)** J774A.1 macrophages or BMDM were primed with 1 μg/ml LPS for 4 h and then treated with YVAD-CHO **(A)** or MCC950 **(B)** for 0.5 h. The cells were then infected with 50 MOI *S. sonnei* for an additional 20 h. The levels of IL-1β in the supernatants were measured by ELISA. **(C,D)** Wild-type or NLRP3 knockout J774A.1 macrophages were primed with 1 μg/ml LPS for 4 h and then infected with 50 MOI *S. sonnei* for an additional 20 h. The levels of IL-1β **(C)** and caspase-1 **(D)** in the supernatants were measured by ELISA and Western blotting, respectively. The ELISA data are expressed as the mean ± SD of four separate experiments. The Western blotting results are representative of three different experiments. * and *** indicate significant differences at the levels of *p* < 0.05 and *p* < 0.001, respectively, compared to *S. sonnei*-infected cells **(A,B)** or untreated control cells **(C)** (one-way ANOVA with Dunnett's multiple comparisons test).

To investigate whether *S. sonnei*-induced IL-1β secretion requires the activation of the NLRP3 inflammasome, LPS-primed J774A.1 macrophages were incubated for 0.5 h with the caspase-1 inhibitor YVAD-CHO before *S. sonnei* infection. We found that YVAD-CHO reduced IL-1β secretion in a dose-dependent manner (Figure 2A). In addition, the NLRP3-specific inhibitor MCC950 reduced *S. sonnei*-induced IL-1β secretion in LPS-primed J774A.1 macrophages and BMDM, indicating the important role of NLRP3 in IL-1β secretion (Figure 2B). The role of NLRP3 in *S. sonnei*-mediated IL-1β secretion was confirmed in J774A.1 macrophages by CRISPR/Cas9-mediated NLRP3 knockout, as *S. sonnei* and ATP failed to induce IL-1β secretion (Figure 2C) and caspase-1 activation in LPS-primed NLRP3 knockout cells (Figure 2D). These results demonstrate that *S. sonnei* induces IL-1β secretion through the NLRP3 inflammasome.

### *S. sonnei* Activates the NLRP3 Inflammasome Through P_2_X_7_ Receptor-Mediated Potassium Efflux

**Figure 3:**
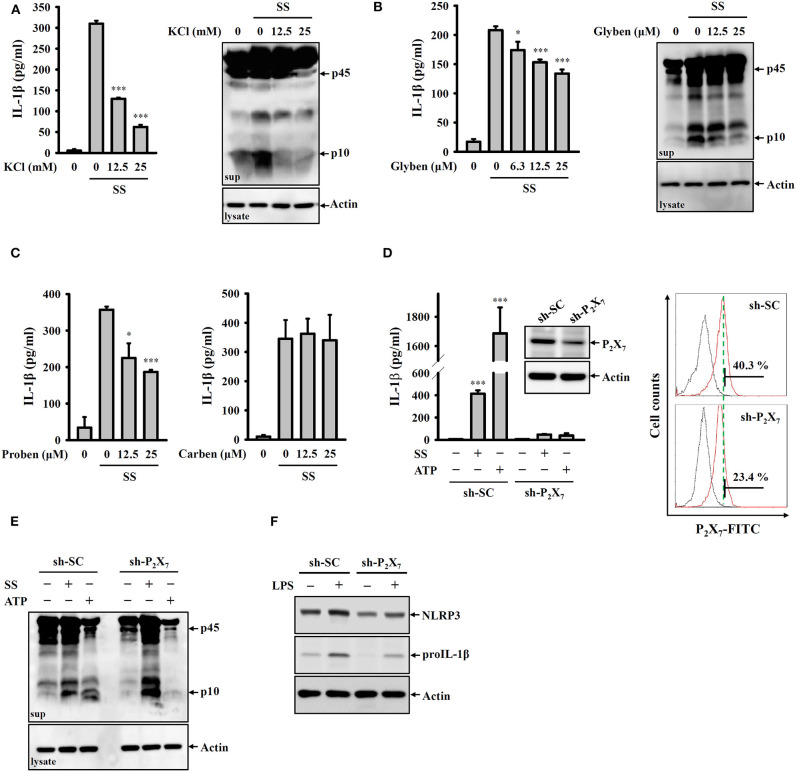
*S. sonnei* activates the NLRP3 inflammasome through P_2_X_7_ receptor-mediated potassium efflux. **(A–C)** J774A.1 macrophages were primed with 1 μg/ml LPS for 4 h and then treated with KCl **(A)**, glibenclamide **(B)**, probenecid or carbenoxolone **(C)** for 0.5 h. The cells were then infected with 50 MOI *S. sonnei* for an additional 20 h. The levels of IL-1β and caspase-1 in the supernatants were measured by ELISA and Western blotting, respectively. **(D,E)** Mock or P_2_X_7_ knockdown J774A.1 macrophages were primed with 1 μg/ml LPS for 4 h and then infected with 50 MOI *S. sonnei* for an additional 20 h or stimulated with 5 mM ATP for an additional 0.5 h. The levels of IL-1β **(D)** and caspase-1 **(E)** in the supernatants were measured by ELISA and Western blotting, respectively. The levels of P_2_X_7_ in mock or P_2_X_7_ knockdown J774A.1 macrophages were measured by Western blotting and flow cytometry **(D)**. **(F)** Mock or P_2_X_7_ knockdown J774A.1 macrophages were stimulated with 1 μg/ml LPS for 6 h. The levels of NLRP3 and proIL-1β in the cell lysates were measured by Western blotting. The ELISA data are expressed as the mean ± SD of four separate experiments. The Western blotting results are representative of three different experiments. * and *** indicate significant differences at the levels of *p* < 0.05 and *p* < 0.001, respectively, compared to *S. sonnei*-infected cells **(A–C)** or untreated control cells **(D)** (one-way ANOVA with Dunnett's multiple comparisons test).

Potassium efflux plays important roles in NLRP3 inflammasome activation in response to various NLRP3 stimulators (28). To investigate whether *S. sonnei* mediates NLRP3 inflammasome activation through potassium efflux, high extracellular potassium concentrations (12.5 and 25 mM KCl in the culture medium) were used to block potassium efflux. We found that *S. sonnei*-mediated IL-1β secretion and caspase-1 activation in J774A.1 macrophages were significantly inhibited by extracellular KCl (Figure 3A). To confirm the role of potassium efflux in *S. sonnei*-mediated NLRP3 inflammasome activation, potassium efflux was blocked by the adenosine triphosphate-sensitive potassium channel blocker glibenclamide. We found that *S. sonnei*-mediated IL-1β secretion and caspase-1 activation in J774A.1 macrophages were significantly inhibited by glibenclamide in a dose-dependent manner (Figure 3B). To further determine the receptor involved in *S. sonnei*-mediated potassium efflux, the effects of probenecid (a P_2_X_7_ receptor inhibitor) and carbenoxolone (a pannexin-1 inhibitor) on *S. sonnei*-induced NLRP3 inflammasome activation were investigated. We found that the P_2_X_7_ receptor inhibitor probenecid, but not the pannexin-1 inhibitor carbenoxolone, reduced IL-1β secretion in *S. sonnei*-infected J774A.1 macrophages (Figure 3C). To provide direct evidence of the important role of the P_2_X_7_ receptor in *S. sonnei*-mediated NLRP3 inflammasome activation, we knocked down the P_2_X_7_ receptor in J774A.1 macrophages by shRNA technology (26). Although the cell surface expression of P_2_X_7_ receptor was partially reduced by shRNA, *S. sonnei*-, and ATP-mediated IL-1β secretion was significantly reduced in P_2_X_7_ receptor knockdown cells compared to scramble shRNA-treated control cells (Figure 3D). Notably, although ATP-mediated caspase-1 activation was significantly reduced in P_2_X_7_ receptor knockdown cells, *S. sonnei*-mediated caspase-1 activation was not reduced in P_2_X_7_ receptor knockdown cells compared to control cells (Figure 3E). To understand why P_2_X_7_ receptor knockdown reduced IL-1β secretion without affecting caspase-1 activation, we investigated the expression levels of NLRP3 and proIL-1β in LPS-activated control and P_2_X_7_ receptor knockdown J774A.1 macrophages. We found that LPS-induced NLRP3 and proIL-1β expression was reduced in P_2_X_7_ receptor knockdown cells (Figure 3F). These results indicate that the P_2_X_7_ receptor participates in the priming of the NLRP3 inflammasome.

### *S. sonnei* Activates the NLRP3 Inflammasome Through H_2_O_2_ Production and Lysosomal Damage

**Figure 4:**
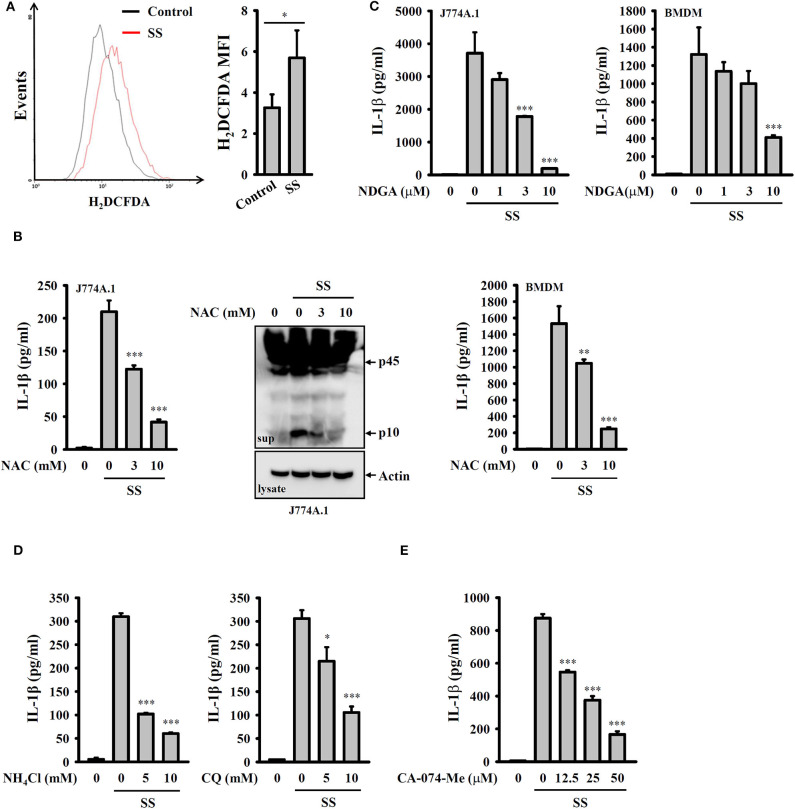
*S. sonnei* activates the NLRP3 inflammasome through H_2_O_2_ production and lysosomal damage. **(A)** J774A.1 macrophages were primed with 1 μg/ml LPS for 4 h and then infected with 50 MOI *S. sonnei* for an additional 20 h. The levels of intracellular ROS were measured by CM-H_2_DCFDA staining, and the data were acquired by flow cytometry. **(B)** J774A.1 macrophages or BMDM were primed with 1 μg/ml LPS for 4 h and then treated with NAC for 0.5 h. The cells were then infected with 50 MOI *S. sonnei* for an additional 20 h. The levels of IL-1β and caspase-1 in the supernatants were measured by ELISA and Western blotting, respectively. **(C)** J774A.1 macrophages or BMDM were primed with 1 μg/ml LPS for 4 h and then treated with NDGA for 0.5 h. The cells were then infected with 50 MOI *S. sonnei* for an additional 20 h. The levels of IL-1β in the supernatants were measured by ELISA. **(D,E)** J774A.1 macrophages were primed with 1 μg/ml LPS for 4 h and then treated with NH_4_Cl and CQ **(D)** or CA-074-Me **(E)** for 0.5 h. The cells were then infected with 50 MOI *S. sonnei* for an additional 20 h. The levels of IL-1β in the supernatants were measured by ELISA. The ELISA data are expressed as the mean ± SD of four separate experiments. The flow cytometry and Western blotting results are representative of three different experiments. *, **, and *** indicate significant differences at the levels of *p* < 0.05, *p* < 0.01 and *p* < 0.001, respectively, compared to untreated control cells **(A)** or *S. sonnei*-infected cells **(B–E)** [two-tailed *t*-test in panel **(A)**; one-way ANOVA with Dunnett's multiple comparisons test in panels **(B–E)**].

It has been proposed that ROS are crucial elements for NLRP3 inflammasome activation (29). We investigated whether *S. sonnei* infection activates the NLRP3 inflammasome through the generation of ROS. We found that *S. sonnei* infection increased H_2_O_2_ production in LPS-primed J774A.1 macrophages, as analyzed by H_2_DCFDA staining (Figure 4A). The inhibition of H_2_O_2_ by the antioxidant NAC reduced IL-1β secretion and caspase-1 activation in *S. sonnei*-infected J774A.1 macrophages (Figure 4B). NAC also reduced IL-1β secretion in *S. sonnei*-infected BMDM (Figure 4B). In addition, the inhibition of ROS generation enzyme lipoxygenase by NDGA reduced IL-1β secretion in *S. sonnei*-infected J774A.1 macrophages and BMDM (Figure 4C), confirming the importance of ROS in *S. sonnei*-mediated IL-1β secretion. Furthermore, the inhibition of the lysosomal cysteine protease cathepsin B by CA-074-me attenuates NLRP3 inflammasome activation in *Mycobacterium tuberculosis*- and *Neisseria gonorrhoeae*-infected cells (30, 31), suggesting an important role for lysosomes in bacterial infection-mediated NLRP3 inflammasome activation. As cathepsin B can be released from damaged lysosomes to drive NLRP3 inflammasome activation through binding to NLRP3 (32), NH_4_Cl, and CQ, which both inhibit endosomal/lysosomal acidification, were used to block lysosomal damage. We found that both NH_4_Cl and CQ significantly reduced IL-1β secretion in *S. sonnei*-infected J774A.1 macrophages (Figure 4D), confirming the role of lysosomes in NLRP3 inflammasome activation in response to *S. sonnei* infection. Furthermore, the cathepsin B inhibitor CA-074-me reduced IL-1β secretion in *S. sonnei*-infected J774A.1 macrophages (Figure 4E). These results indicate that *S. sonnei* activates the NLRP3 inflammasome through H_2_O_2_ production and lysosomal damage.

### *S. sonnei* Activates the NLRP3 Inflammasome Through Mitochondrial Damage

**Figure 5:**
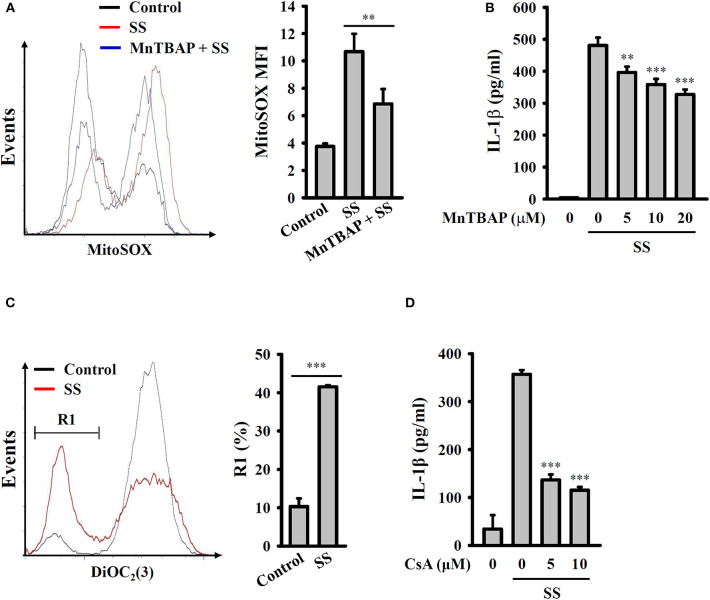
*S. sonnei* activates the NLRP3 inflammasome through mitochondrial damage. **(A)** J774A.1 macrophages were primed with 1 μg/ml LPS for 4 h and then treated with 20 μM MnTBAP for 0.5 h. The cells were then infected with 50 MOI *S. sonnei* for an additional 20 h. The levels of mitochondrial ROS were measured by MitoSOX staining and flow cytometry. **(B)** J774A.1 macrophages were primed with 1 μg/ml LPS for 4 h and then treated with MnTBAP for 0.5 h. The cells were then infected with 50 MOI *S. sonnei* for an additional 20 h. The levels of IL-1β in the supernatants were measured by ELISA. **(C)** J774A.1 macrophages were primed with 1 μg/ml LPS for 4 h and then infected with 50 MOI *S. sonnei* for an additional 20 h. The mitochondrial membrane potential was measured by DiOC_2_(3) staining and flow cytometry. **(D)** J774A.1 macrophages were primed with 1 μg/ml LPS for 4 h and then treated with cyclosporine A for 0.5 h. The cells were then infected with 50 MOI *S. sonnei* for an additional 20 h. The levels of IL-1β in the supernatants were measured by ELISA. The ELISA data are expressed as the mean ± SD of four separate experiments. The flow cytometry results are representative of three different experiments. ** and *** indicate significant differences at the levels of *p* < 0.01 and *p* < 0.001, respectively, compared to *S. sonnei*-infected cells **(B,D)** or as indicated **(A,C)** [two-tailed *t*-test in panels **(A,C)**; one-way ANOVA with Dunnett's multiple comparisons test in panels **(B,D)**].

It has been demonstrated that mitochondrial damage drives downstream signaling that leads to NLRP3 inflammasome activation (33). Mitochondrial ROS are one downstream signals of damaged mitochondria that activate the NLRP3 inflammasome. Using the mitochondrial ROS indicator MitoSOX, we demonstrated that *S. sonnei* infection significantly induced mitochondrial ROS production in J774A.1 macrophages and that this effect was inhibited by MnTBAP, a mimic of superoxide dismutase (Figure 5A). We also found that the inhibition of mitochondrial ROS production by MnTBAP inhibited IL-1β secretion in *S. sonnei*-infected J774A.1 macrophages (Figure 5B). In addition, we found that *S. sonnei* infection caused mitochondrial integrity loss in J774A.1 macrophages, as analyzed by the mitochondrial membrane-potential-sensitive probe DiOC_2_(3) (Figure 5C). Furthermore, preserving mitochondrial integrity with cyclosporine A, an inhibitor of mitochondrial membrane permeability transition (33), reduced IL-1β secretion in *S. sonnei*-infected J774A.1 macrophages (Figure 5D). These results suggest that *S. sonnei* activates the NLRP3 inflammasome through mitochondrial damage.

### Phagocytosis of Live *S. sonnei* Is Required for the Full Activation of the NLRP3 Inflammasome

**Figure 6:**
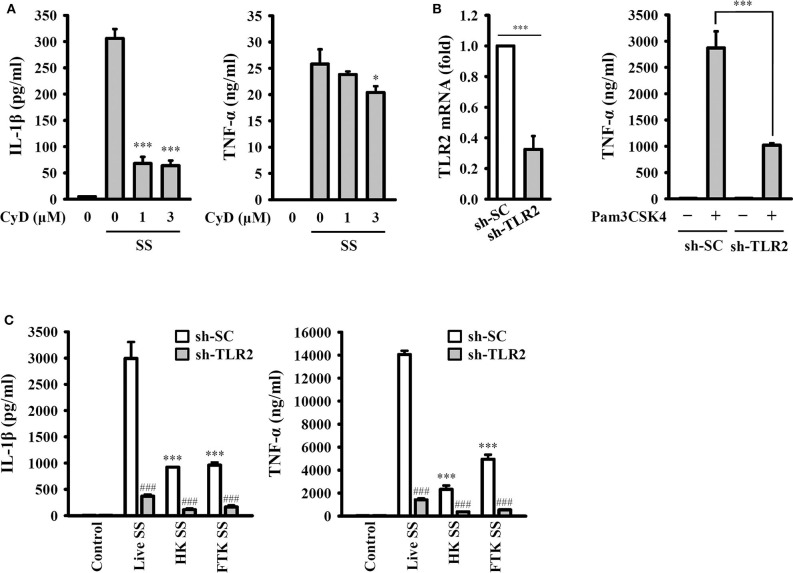
Phagocytosis of live *S. sonnei* is required for the full activation of the NLRP3 inflammasome. **(A)** J774A.1 macrophages were primed with 1 μg/ml LPS for 4 h and then treated with cytochalasin D for 0.5 h. The cells were then infected with 50 MOI *S. sonnei* for an additional 20 h. The levels of IL-1β and TNF-α in the supernatants were measured by ELISA. **(B)** The TLR2 mRNA expression of mock or TLR2 knockdown J774A.1 macrophages was measured by RT-qPCR. Mock or TLR2 knockdown J774A.1 macrophages were stimulated with 1 μg/ml Pam3CSK4 for 6 h. The levels of TNF-α in the supernatants were measured by ELISA. **(C)** Mock or TLR2 knockdown J774A.1 macrophages were primed with 1 μg/ml LPS for 4 h and then infected with 50 MOI live, heat-killed or freeze/thaw-killed *S. sonnei* for an additional 20 h. The levels of IL-1β and TNF-α in the supernatants were measured by ELISA. The ELISA data are expressed as the mean ± SD of four separate experiments. * and *** indicate significant differences at the levels of *p* < 0.05 and *p* < 0.001, respectively, compared to *S. sonnei*-infected cells **(A)** or live *S. sonnei*-infected cells **(C)** or as indicated **(B)**. ^*###*^indicates significant differences at the levels of *p* < 0.001 compared to sh-SC cells [one-way ANOVA with Dunnett's multiple comparisons test in panels **(A,C)**; two-tailed *t*-test in panel **(B)**].

To investigate whether the activation of the NLRP3 inflammasome requires phagocytosis of *S. sonnei*, J774A.1 macrophages were incubated with cytochalasin D, a cell-permeable actin polymerization inhibitor, which blocks the phagocytosis of macrophages. We found that cytochalasin D significantly reduced IL-1β secretion but only slightly reduced TNF-α secretion in *S. sonnei*-infected J774A.1 macrophages (Figure 6A). These results indicate that phagocytosis of *S. sonnei* is required for the full activation of the NLRP3 inflammasome but plays a smaller role in NLRP3-independent TNF-α secretion. In addition, our previous study indicated that TLR2 and phagocytosis of live *Neisseria gonorrhoeae* are important for the NLRP3 inflammasome activation (26). To investigate the role of TLR2 and live *S. sonnei* in NLRP3 inflammasome activation, we generated TLR2 knockdown J774A.1 macrophages by shRNA technology (26). We found that TLR2 mRNA expression was significantly reduced in sh-TLR2 cells compared to the scramble control (sh-SC) cells, and TLR2 ligand Pam3CSK4-mediated TNF-α secretion was significantly reduced in sh-TLR2 cells compared to sh-SC cells, indicating the functional knockdown of TLR2 in the cells (Figure 6B). We investigated the effect of live, heat-killed, or freeze/thaw-killed *S. sonnei* on IL-1β and TNF-α in sh-TLR2 and sh-SC cells. We found that heat-killed and freeze/thaw-killed *S. sonnei* induced less IL-1β and TNF-α secretion than that induced by live *S. sonnei* (Figure 6C). These results indicate that the NLRP3 inflammasome activation and TNF-α expression requires phagocytosis of live *S. sonnei*. In addition, we found that live, heat-killed, and freeze/thaw-killed *S. sonnei* induced less IL-1β and TNF-α secretion in sh-TLR2 cells than that in sh-SC cells (Figure 6C). These results indicate that TLR2 plays an important role in *S. sonnei*-mediated IL-1β and TNF-α secretion in macrophages.

### NLRP3 Knockout Increases the Bactericidal Activity of Macrophages Against *S. sonnei*

**Figure 7:**
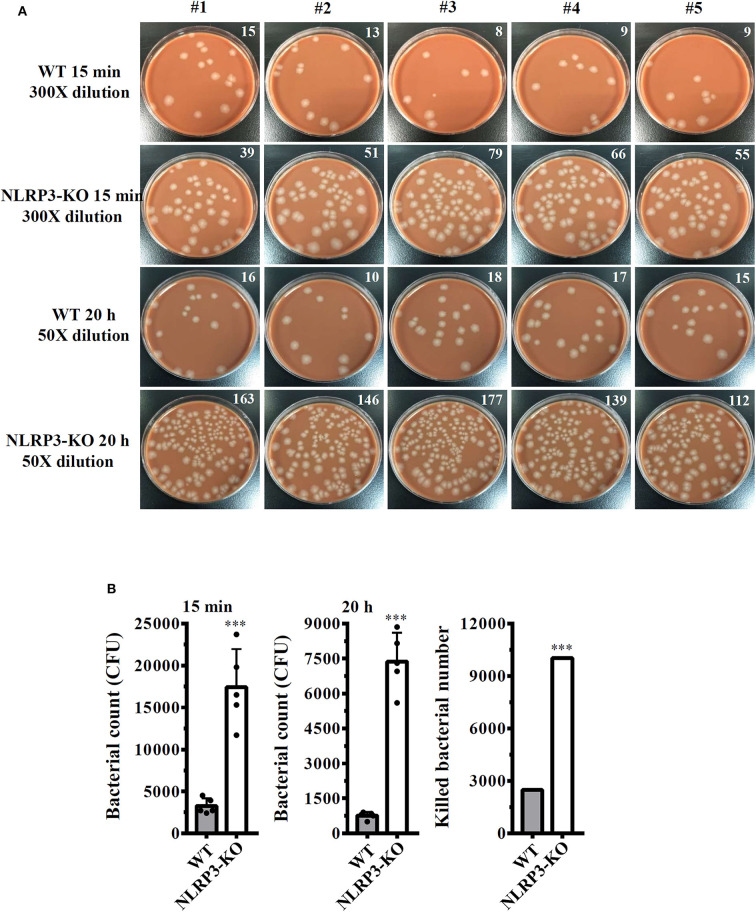
NLRP3 knockout increases the bactericidal activity of macrophages against *S. sonnei*. **(A,B)** Wild-type or NLRP3-knockout J774A.1 macrophages were infected with 50 MOI *S. sonnei* for 15 min or 20 h. The cells were lysed, and the number of engulfed live *S. sonnei* cells was determined by the CFU assay and is indicated in the upper right corner **(A)**. The mean CFUs of cells infected with *S. sonnei* for 15 min or 20 h and the number of killed bacteria were calculated by subtracting the 20 h CFU assay result from the 15 min CFU assay result and are shown in panel **(B)**. The data are expressed as the mean ± SD of five separate experiments. ***indicates a significant difference at the level of *p* < 0.001 compared to wild-type cells (two-tailed *t-*test).

Our previous study demonstrated that the knockout of NLRP3 by CRISPR/Cas9 technology enhances phagocytosis of pHrodo Green *E. coli* BioParticles Conjugate and *Neisseria gonorrhoeae* by macrophages (26). In this study, we assessed the functional consequence of NLRP3 knockout on phagocytosis of *S. sonnei* by macrophages. The number of engulfed bacteria in wild-type and NLRP3 knockout J774A.1 macrophages was determined by the CFU assay after 15 min of infection. The extracellular bacteria were washed out and completely killed by gentamicin. We found that the number of engulfed bacteria in NLRP3 knockout cells (17,400 ± 1,665 CFUs) was higher than that in wild-type cells (3,240 ± 332 CFUs; Figures 7A,B). These results indicated that NLRP3-knockout increased phagocytosis of *S. sonnei* by macrophages. To further investigate the functional consequence of NLRP3 knockout on the bactericidal activity of macrophages against *S. sonnei*, the number of intracellular live *S. sonnei* cells after 20 h of infection was determined by the CFU assay. We found that the number of intracellular live *S. sonnei* cells in NLRP3 knockout cells and wild-type cells was 7,370 ± 451 and 760 ± 57, respectively (Figures 7A,B). The number of killed *S. sonnei* cells was calculated by subtracting the 20 h CFU assay result from the 15 min CFU assay result. We found that NLRP3 knockout significantly increased the bactericidal activity of macrophages against *S. sonnei* (10,030 *S. sonnei* cells were killed) compared to wild-type cells (2,480 *S. sonnei* cells were killed; Figure 7B). These results indicate that NLRP3 knockout increases the phagocytic and bactericidal activity of macrophages against *S. sonnei*.

## Discussion

Inflammation is a process of the immune system that regulates the host defense machinery and controls microbial invasion; however, over-reactive or prolonged inflammation increases the risk for the development of inflammatory diseases. Precise control of inflammatory responses is important for limiting pathogen infection without causing host damage. The NLRP3 inflammasome controls the maturation and secretion of the proinflammatory cytokines IL-1β and IL-18 and is important for the innate immunity against pathogen infection (12, 26). The dysregulation of NLRP3 inflammasome activation has been demonstrated to participate in the pathogenesis of metabolic disorders and neurodegenerative diseases (15, 34). The findings of this study clearly showed that *S. sonnei* infection caused IL-1β and IL-18 production through the NLRP3 inflammasome in macrophages, suggesting that *S. sonnei* infection may increase the risk for NLRP3-associated inflammatory diseases, including diabetes, atherosclerosis, inflammatory bowel disease, chronic kidney disease, gout, and Alzheimer's disease (13–15).

The activation of the NLRP3 inflammasome is initiated by 2-step priming and activating signals. The priming step involves pathogen-associated molecular patterns (e.g., LPS) through toll-like receptors to induce the protein expression of NLRP3 and the IL-1β precursor (25). The activating step involves a broad range medically relevant stimuli, including saturated fatty acids (type II diabetes), cholesterol crystals (atherosclerosis), uric acid crystals (gouty inflammation), and amyloid-β (Alzheimer's disease) (15). In this study, we demonstrated that *S. sonnei* infection did not provide the priming signal of the NLRP3 inflammasome because *S. sonnei* infection induced IL-1β secretion in LPS-primed macrophages but not in macrophages without LPS priming (Figure 1A). However, other studies have shown that *S. flexneri* (strain YSH6000) infection induces IL-1β secretion in bone marrow-derived macrophages without LPS priming (20, 22). These differences may come from the different serotypes of *Shigella* or cell types. The activating signals of the NLRP3 inflammasome include extracellular ATP, bacterial pore-forming toxins, and crystal substances that induce ROS production, ion efflux, lysosomal damage, and mitochondria stress (15). Finally, the NLRP3 protein recruits adaptor protein ASC to form oligomers, activate caspase-1, and induce the maturation of IL-1β and IL-18 (34, 35). It has been demonstrated that invasion plasmid antigen H7.8 enzyme 3 ubiquitin ligase is important for *S. flexneri*-mediated NLRP3 and NLR family CARD domain containing 4 (NLRC4) inflammasome activation, as it digests the NLRP3 and NLRC4 inflammasome inhibitory proteins glomulin/flagellar-associated protein 68 (22, 36). Furthermore, needle- or rod-shapes proteins secreted by the *S. flexneri* type III secretion system are recognized by NAIP1 and NAIP2 to induce robust NLRC4 inflammasome activation (37–40). RAW264.7 macrophages infected with *S. flexneri* induce NLRP1B inflammasome activation (41). This study was limited because it did not identify the virulence factor of *S. sonnei* for NLRP3 inflammasome activation. We demonstrated that, compared to live *S. sonnei*, killed *S. sonnei* significantly reduced the IL-1β induction activity (Figure 6C). In addition, a phagocytosis inhibitor also significantly reduced IL-1β induction in *S. sonnei*-infected macrophages (Figure 6A). These results indicate that the intracellular active delivery of the virulence factor by *S. sonnei* is important for NLRP3 inflammasome activation. Recent study demonstrated that *S. sonnei* infection caused caspase-1 activation, ASC speck formation, and IL-18 expression in THP-1 macrophages. However, *S. sonnei* infection caused less caspase-1 dependent pyroptosis of macrophages than *S. flexneri* infection. In a mechanistic study, the O-antigen on the surface of *S. sonnei* reduced bacterial uptake and cytosolic escape, which results in reduced activation of caspase-1 (42).

The P_2_X_7_ receptor is a cation-specific ion channel that recognizes and responds to extracellular ATP and induces potassium efflux. the activation of the P_2_X_7_ receptor is associated with the immune response and regulates pathogen infection (43). In this study, we demonstrated that the P_2_X_7_ receptor participated in IL-1β release in *S. sonnei*-infected macrophages (Figure 3D). Notably, although P_2_X_7_ receptor knockdown significantly reduced caspase-1 activation in ATP-activated macrophages, caspase-1 activation was not affected in *S. sonnei*-infected macrophages (Figure 3E). One explanation for is P_2_X_7_ receptor knockdown-mediated IL-1β inhibition in *S. sonnei*-infected macrophages is the reduced expression of NLRP3 and proIL-1β (Figure 3F). These results indicated that P_2_X_7_ receptor knockdown reduced NLRP3 inflammasome activation by inhibiting the priming signal but not by affecting the activation signal in *S. sonnei*-infected macrophages. It should be noted that only partial inhibition of P_2_X_7_ receptor with shRNA, but total inhibition of IL-1β in *S. sonnei*-infected macrophages (Figure 3D). It has been demonstrated that stimulation of P_2_X_7_ receptor activates caspase-1 dependent pyroptosis and induces the formation of a non-selective pore, allowing the release of intracellular components including IL-1β (44). We suggested that P_2_X_7_ receptor knockdown not only reduced the proIL-1β expression but also inhibited the IL-1β release by reducing pyroptosis. A previous study demonstrated that the P_2_X_7_ receptor positively regulates LPS-induced TNF-α secretion by increasing the extracellular activity of the TNF-α converting enzyme (45), indicating that the P_2_X_7_ receptor can regulate LPS-mediated proinflammatory signaling. The inhibition of the P_2_X_7_ receptor not only reduces IL-1β production by *S. sonnei*-infected macrophages but also reduces IL-1β production by *Neisseria gonorrhoeae*- and Group A *Streptococcus*-infected macrophages (26, 46). These results suggest that the P_2_X_7_ receptor is a potential therapeutic target for pathogen infection (47, 48) or inflammatory diseases (49).

In this study, we demonstrated that *S. sonnei* infection induces inflammatory responses by activating the NLRP3 inflammasome. We also found that NLRP3 knockout enhanced the phagocytic and bactericidal activity of macrophages against *S. sonnei* (Figure 7). This study provides a rational strategy for protecting against *S. sonnei* infection and reducing inflammatory damage by targeting the NLRP3 inflammasome. In addition, the *in vitro S. sonnei* infection model can be used to screen and develop potential compounds or ingredients to ameliorate *S. sonnei*-induced inflammation. However, limitation of this study is the lack of an animal model of *S. sonnei* infection. Because of the complications of *Shigella* pathogenesis, there is no ideal model animal model that faithfully recapitulates *Shigella* infection in humans. Recently, Koestler et al. (50) cultured human intestinal stem cell-derived intestinal enteroids *ex vivo* and mimicked the human intestinal environment. The effect of *S. sonnei* infection on the NLRP3 inflammasome can be confirmed in the human intestinal enteroid model in the future.

## Data Availability Statement

The datasets generated for this study are available on request to the corresponding author.

## Author Contributions

K-FH was the guarantor of the article. L-HL and K-FH conceived and designed the study, wrote, and finished the manuscript. L-HL, T-LC, H-WC, and T-TT performed the experiments and analyzed the data. C-HH, C-CW, and W-CT contributed to critical revision of the manuscript. T-CJ, F-HC, and OC assisted with some experiments.

## Conflict of Interest

The authors declare that the research was conducted in the absence of any commercial or financial relationships that could be construed as a potential conflict of interest.
